# Idiopathic Myointimal Hyperplasia of the Mesenteric Veins: A Rare Mimic of Inflammatory Bowel Disease

**DOI:** 10.7759/cureus.81295

**Published:** 2025-03-27

**Authors:** Rodrigo Cañada T Surjan, Paulo A Laginha, Raquel B Ferreira, Ryan Y Tanigawa, Jose C Ardengh

**Affiliations:** 1 Digestive Surgery, Faculdade de Medicina da Universidade de São Paulo, São Paulo, BRA; 2 Surgery, HCOR - Associação Beneficente Síria, São Paulo, BRA; 3 Medicine and Surgery, UNISA - Universidade Santo Amaro, São Paulo, BRA; 4 Pathology, HCOR - Sociedade Beneficente Siria, São Paulo, BRA; 5 Endoscopy, Hospital Moriah, São Paulo, BRA

**Keywords:** abdominal pain differential diagnosis, idiopathic myointimal hyperplasia, inflammatory bowel disease, intestinal bleeding, intestinal ischemia, partial colectomy

## Abstract

Idiopathic myointimal hyperplasia of the mesenteric veins (IMHMV) is a rare cause of segmental intestinal ischemia. It is usually associated with chronic symptoms of abdominal pain, diarrhea, and rectal bleeding, but it also can present in more acute and dramatic clinical presentations. Surgical segmental bowel resection is the proper treatment, but it is commonly delayed by the misdiagnosis of this condition as inflammatory bowel disease, such as Crohn's or idiopathic ulcerative colitis. Awareness of IMHMV as a non-thrombotic cause of intestinal ischemia may prevent unnecessary medical treatment with topical and systemic steroids, antibiotics, and even mesalazine and avoid potentially fatal complications of the disease. We report a case with early development of severe complications and also review the literature on IMHMV.

## Introduction

Idiopathic myointimal hyperplasia of the mesenteric veins (IMHMV) was first described by Genta and Haggitt in 1991 in a series of four patients as a non-thrombotic cause of intestinal ischemia [1]. Different from other ischemic conditions often caused by arterial thromboembolism or venous thrombosis, IMHMV is caused by a proliferation of smooth muscle in the tunica intima of vein walls [2].

Middle-aged males are predominantly affected (80%, mean age 58 years), and the sigmoid colon is the segment most affected (79%) [3,4]. It is associated with chronic abdominal pain (94%), diarrhea (71%), unintentional weight loss (25%), and hematochezia (67%) and is frequently misdiagnosed as inflammatory bowel disease (IBD) [3,4]. Final diagnosis usually comes after pathological examination of resected specimens during surgery for complications (such as extensive bleeding).

Although there are a few theories for the pathophysiology of IMHMV, often suggesting arteriovenous fistulas as a result of repetitive traumas of intermittent volvulus or torsion of hypermobile sigmoid colon, the precise pathophysiology of IMHMV is unknown [4].

Due to similarities in clinical presentation (such as abdominal pain, diarrhea, hematochezia) and on image studies findings such as thickening of the intestinal walls on abdominal computed tomography or mucosal edema with erythema and cobblestone appearance of the intestinal mucosa on colonoscopy, IMHMV is usually misdiagnosed as IBD, including ulcerative colitis and Crohn's disease. Therefore, preoperative diagnosis of IMHMV is challenging. As a result, proper treatment with upfront surgery is rarely performed, and unnecessary medical treatment is usually the initial choice [5]. Moreover, as IMHMV is a progressive disease, approximately 30% of the patients may experience severe complications as a result of diagnostic delay [6].

We report a case of IMHMV in a 46-year-old male who presented mainly with intense lower abdominal pain and rectal bleeding that was submitted initially to medical treatment for a suspected IBD. Less than a month after the onset of symptoms, he presented severe hemorrhage and was submitted for emergency rectosigmoidectomy. Pathological examination of the surgical specimen allowed a definitive diagnosis of IMHMV. One year after the procedure the patient is asymptomatic and not using medications.

This report emphasizes the importance of the awareness of IMHMV and careful interpretation of diagnostic tests to reach early and preoperative diagnosis in order to avoid unnecessary medical treatment and the risk of severe complications. We also propose a diagnostic and therapeutic algorithm for this rare disease.

## Case presentation

A 46-year-old male, with no comorbidities or significant medical and family history, was referred from another hospital after 2 weeks of hospitalization for intense and refractory-to-opioids pain in the lower abdomen, especially during evacuation and recurrent and significative rectal bleeding. This complaint was initiated 5 days before hospitalization. At arrival, he was taking oral corticosteroids (prednisolone 40 mg orally every 24 hours) and mesalazine orally (3 grams daily) and rectally (enema 4 grams daily) as a form of treatment for potential IBD, after being submitted to an abdominal computed tomography scan and a colonoscopy with biopsies.

At admission to our hospital, the patient was afebrile and hemodynamically stable (heart rate 82 beats/minute and blood pressure 100/60 mm Hg). Physical examination presented lower abdominal pain without signs of peritonitis and rectal exam was extremely painful but without the presence of blood on the tip of the glove.

**Figure 1:**
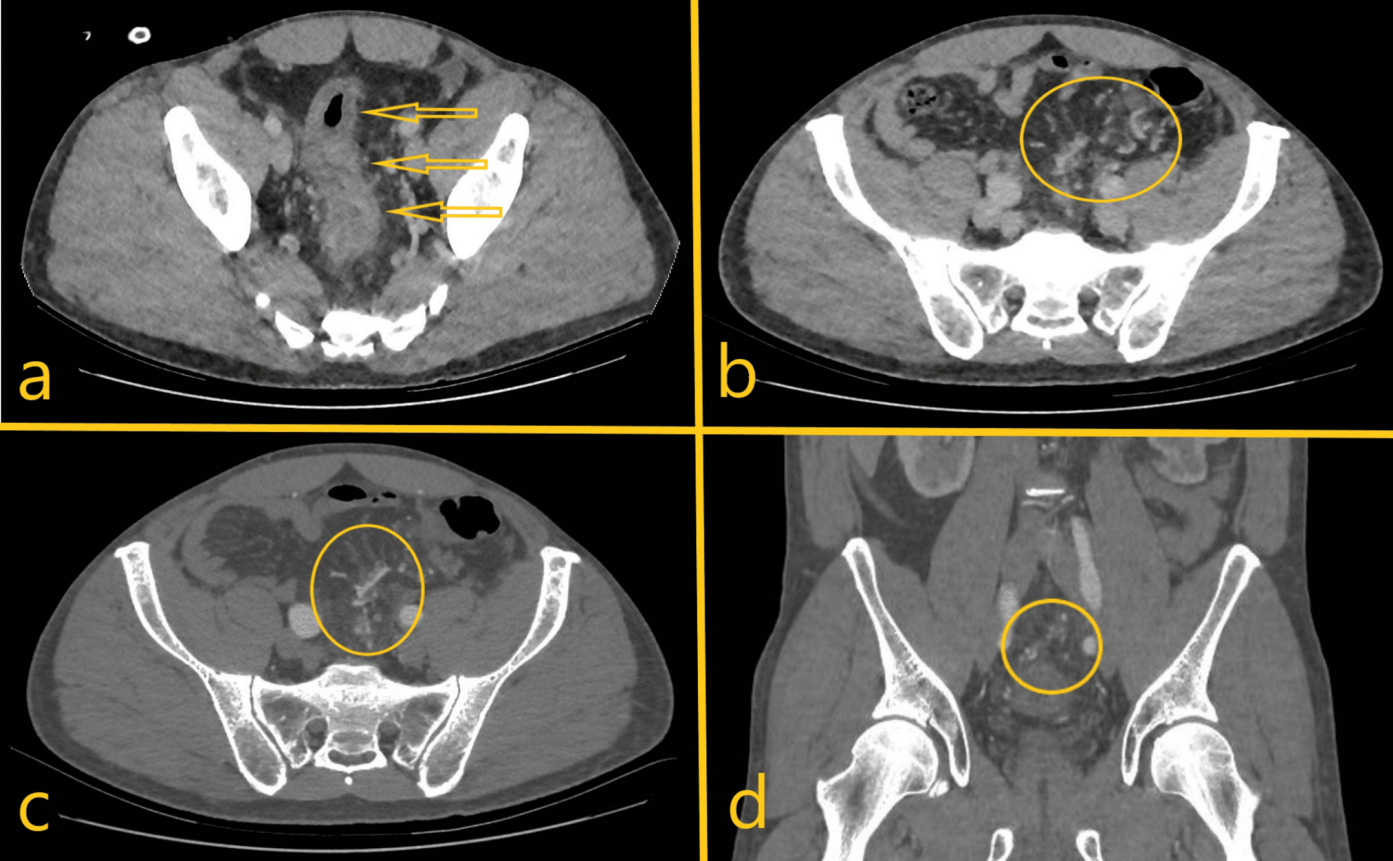
Computed angiotomography axial plane images. a) Thickening of the rectum wall, finding that can be similar to IBD (yellow arrows); b,c) Exuberant vascular engorgement along the walls of the rectum and sigmoid suggestive of angiodysplasia (yellow circles), that can be a suggestive sign of IMHMV; d) Signs of obstruction of the inferior mesenteric vein (yellow circle). IBD: inflammatory bowel disease; IMHMV: Idiopathic myointimal hyperplasia of the mesenteric veins

Complementary imaging exams were repeated at our institution on the day of hospital admission. An abdominal computed angiotomography disclosed parietal thickening of the sigmoid colon and rectum with blurring of the adjacent adipose planes, which may be associated with an inflammatory process, and was interpreted as suggestive of IBD. It also disclosed exuberant vascular engorgement near the rectum and sigmoid wall, with signs of obstruction of the inferior mesenteric vein (Figure 1). 

**Figure 2:**
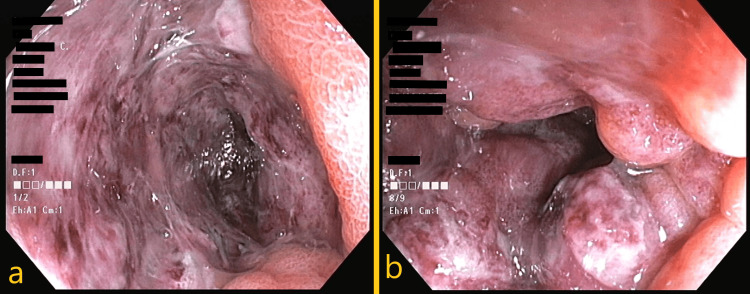
Colonoscopy findings. a) Friable mucosal edema with near-circumferential ulceration, suggestive of inflammatory disease or ischemia, absence of loss of vascular markings that can be present in ulcerative colitis. Notice the continuous aspect of the ulceration, different from Crohn's intervening normal mucosa between ulcers; b) Cobblestone ulcers, also present in many cases of IBD. IBD: inflammatory bowel disease

On hospital day 2, a rectosigmoidoscopy was performed and revealed a very edematous mucosa with circumferential friable areas of violet coloring, affecting the sigmoid and rectum and cobblestone ulcers. In the distal rectum, there were active shallow ulcers covered by fibrin, where biopsies were performed. The findings were suggestive of ischemia and not clearly indicative of active IBD, but a diagnostic hypothesis of IMHMV was not proposed (Figure 2). An upper digestive endoscopy was also performed on the same day that did not disclose any significant findings.

As the biopsies of the rectosigmoidoscopy were non-specific and did not rule IBD, medical treatment with intravenous steroids (methylprednisolone 60 mg every 24 hours), rectal (enema 4 grams daily) and oral (3 grams daily) mesalazine, glutamine (10 grams every 24 hours), large spectrum antibiotics (intravenous ciprofloxacin 400 mg every 12 hours and intravenous metronidazole 500 mg every 8 hours), and symptomatic medications were continued.

**Figure 3:**
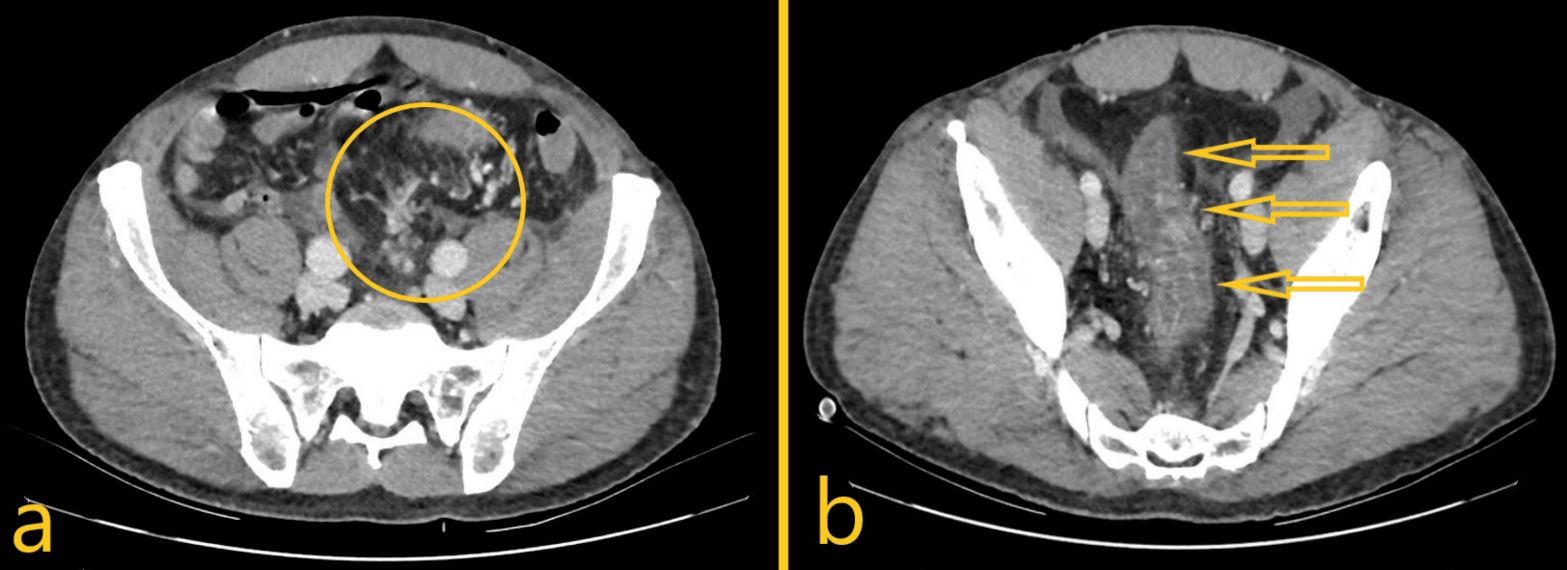
Abdominal computed tomography axial plane images. a) Early opacification of venous branches adjacent to the rectum and sigmoid, suggesting angiodysplasia (yellow circle) and maintained signs of non-thrombotic inferior mesenteric vein obstruction, findings that may be suggestive of other causes of bowel ischemia or inflammatory disease, such as IMHMV or enterocolic lymphocytic phlebitis; b) Persistence of thickening of the rectum and sigmoid walls (yellow arrows) with slight blurring of the surrounding fat, which may represent an inflammatory process. IMHMV: Idiopathic myointimal hyperplasia of the mesenteric veins

For a week, medical treatment was maintained with periods of clinical improvement followed by symptomatic recurrence with intense pelvic pain during evacuation and rectal bleeding episodes. A control abdominal tomography was performed on hospital day 6 and showed no significant image changes (Figure 3).

At this point, the patient was receiving unnecessary medical treatment, and an adequate diagnosis was not reached. As IMHMV is a progressive disease with potentially severe complications, on hospital day 8, less than one month after the onset of symptoms (27 days since symptom onset), the patient presented with intense rectal bleeding with loss of consciousness, hemodynamic instability, and more than 5 g/dL acute hemoglobin drop (from 13.4 to 8.1 g/dL). Initial prompt care was provided in the hospital ward and then he was taken to the surgical center.

**Figure 4:**
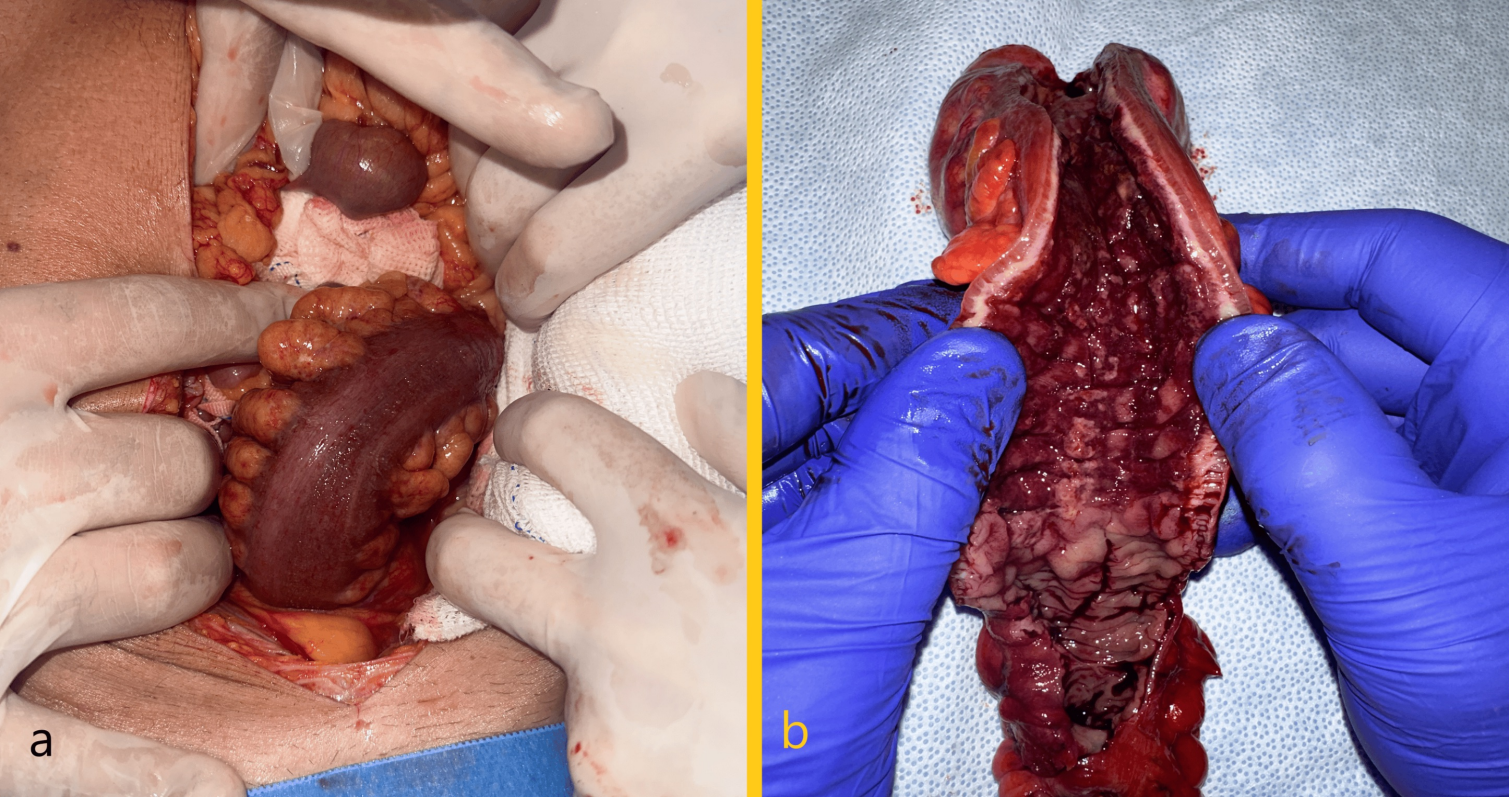
Intraoperative images. a) At initial laparotomy and inspection, ischemic sigmoid colon; b) Non-fixated 20 cm rectosigmoidectomy specimen after resection: cobblestone mucosa, thickening of the intestinal wall.

Due to hemodynamic instability and active intense rectal bleeding, a decision was made to perform a laparotomy. On inspection, we could clearly identify a 20 cm ischemic bowel segment with thickened mesentery corresponding to the rectosigmoid junction and a Hartmann rectosigmoidectomy (Hartmann procedure) was performed. Surgery time was 2 hours, during which he received three bags of red blood cell concentrates (Figure 4). The immediate postoperative period was spent in an intensive care unit. The postoperative period was uneventful, and the patient was discharged on the seventh postoperative day.

**Figure 5:**
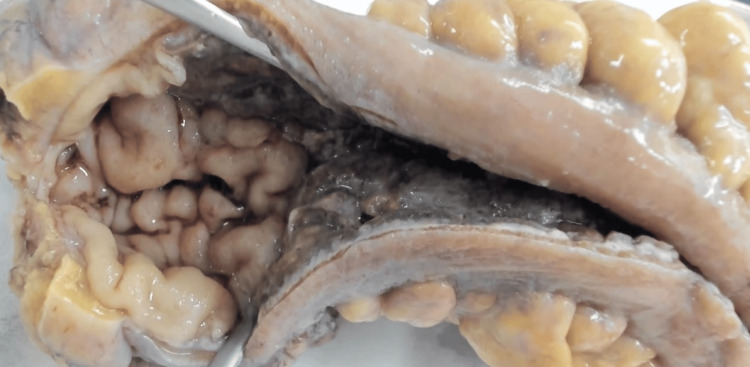
Macroscopic examination of surgical specimen after fixation, with sigmoid wall thickening and cobblestone mucosa, similar to inflammatory bowel disease.

**Figure 6:**
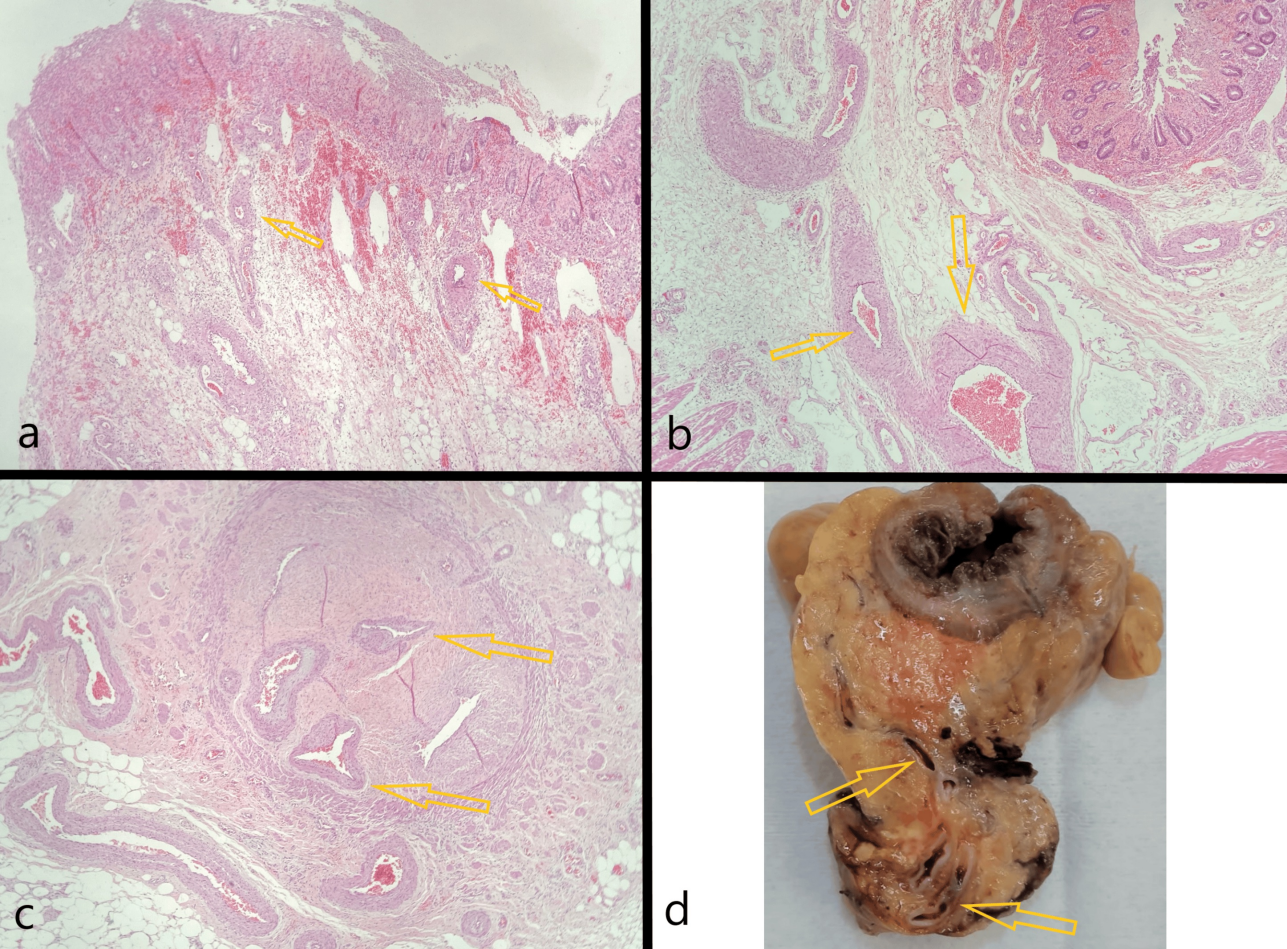
Pathologic examination. a) H&E, x40: ischemic necrosis of the mucosa with venules with thickened walls in the submucosa (yellow arrows); b) H&E, x40: submucosal veins with hypertrophy of the muscular layer (yellow arrows); c) H&E, x100: mesocolic veins with luminal obliteration with muscular hypertrophy (yellow arrows), allowing the diagnosis IMHMV. This pathological finding is not represented on superficial colonoscopy biopsies; d) Mesocolon with thickened venous walls and adipose tissue necrosis. H&E: hematoxylin and eosin; IMHMV: Idiopathic Myointimal Hyperplasia of the Mesenteric Veins

The final diagnosis was reached through pathological examination of the surgical specimen, consistent with IMHMV (Figures 5,6).

**Figure 7:**
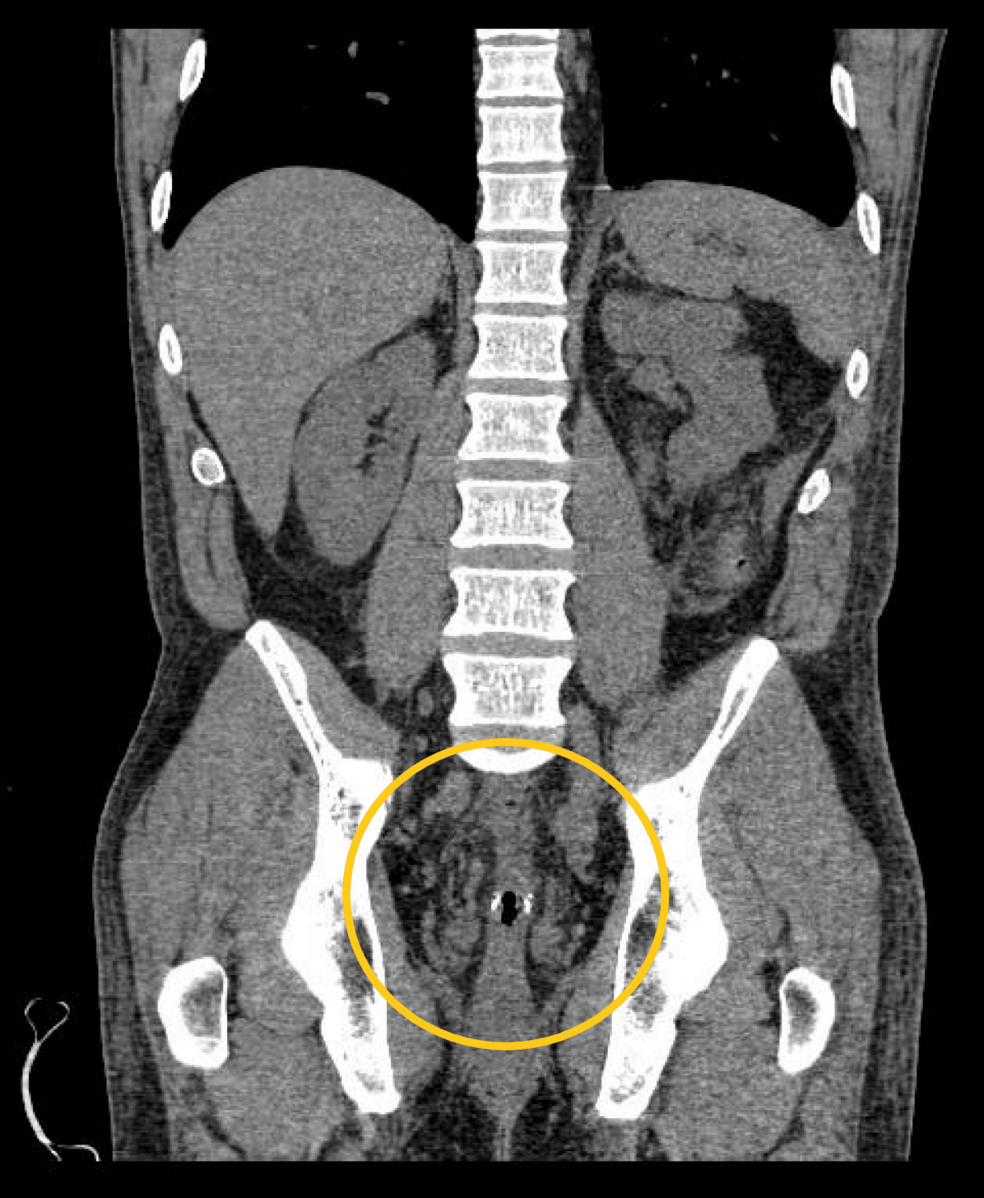
Abdominal computed tomography, coronal plane. Slight thickening of the remaining rectum.

After the procedure, the patient did not develop any abdominal pain or rectal bleeding. An abdominal computed tomography performed 2 weeks after the procedure did not disclose signs of any bowel segment inflammation (Figure 7).

Two months after the Hartmann procedure, the reconstruction was performed uneventfully. One year after the initial symptoms, the patient is asymptomatic and not taking any medication.

## Discussion

IMHMV is a rare cause of non-thrombotic intestinal ischemic injury, with approximately 70 cases reported in English-language literature [7]. However, due to its clinical presentation and image studies' similarity to IBD, it is probable that several other cases were misdiagnosed or not reported. IMHMV usually affects healthy men (80%) at a mean age of 58 years, and in most cases, the sigmoid colon is affected [3,4].

Another important differential diagnosis is also a rare condition - enterocolic lymphocytic phlebitis (ELP). First described in 1976, it consists of lymphocytic inflammation of gastrointestinal veins and venules, and most commonly and unlike IMHMV, it affects the terminal ileum and proximal colon (although it may affect various segments of the small bowel and even the gallbladder) [8]. Symptoms can also mimic IMHMV, including abdominal pain, hematochezia, and diarrhea, and the final diagnosis, as in IMHMV, is usually performed after pathological examination of surgically resected specimens [9]. 

The etiology of IMHMV is unknown, and there is no current evidence of genetic risk factors or environmental triggers. Genta and Haggitt, in their first report of the disease in 1991, raised the hypothesis of arteriovenous fistulas (AVFs) being the cause of this vascular condition [1,7], with similar pathology findings to failed cardiac saphenous veins bypass graft and stenosis of AVFs created to perform dialysis. Abu-Alfa AK et al and Sherman J et al stated that these AVFs could be a result of trauma to the mesocolon such as repeated intermittent torsion because of the augmented mobility of the sigmoid colon [10,11]. Other authors tended to associate IMHMV with comorbidities such as diabetes mellitus, hypertension, primary biliary cirrhosis, hyperlipidemia, or ulcerative colitis [7,10-12].

The symptoms of IMHMV are usually non-specific and chronic (weeks or a few months) and include lower abdominal pain, diarrhea alternating with constipation, weight loss, constipation, and hematochezia. Mostly, the rectum and sigmoid colon are affected, but it can present even as a pancolonic or ileal disease [7].

As in our case, where the disease presented with acute onset and early severe complication with massive rectal bleeding, there are only eight other cases reported in the literature with acute onset. Nevertheless, none of those presented with an episode of acute hemorrhage [7]. Consistent with the literature, our patient was a previously healthy middle-aged male and the sigmoid colon was affected [7]. There is only one death reported in 70 described patients in the literature, as a result of multiple reoperations for anastomotic leakages and perforations in a patient that initially presented with pancolonic and ileal disease [7,13].

Diagnostic exams performed during investigation are most frequently colonoscopy and computed tomography. Colonoscopy findings are associated with ischemic mucosal changes and are usually non-specific, such as ulcerations, and mucosal congestion and friability. Some findings such as continuous ulceration without intervening normal mucosa between ulcers and absence of loss of vascular markings are signs that can be taken into consideration as not suggestive of Crohn's disease or ulcerative colitis, respectively. Colonoscopy biopsies usually are not diagnostic, as they usually are not capable of distinguishing IMHMV from other pathologies such as IBD, and final diagnosis is reached only when surgical specimens disclose the pathognomonic intimal smooth muscle hyperplasia of vessels preserving arterial vessels [7]. Computed tomography findings are also usually non-specific, such as colonic and rectal thickening, poor mural enhancement, and pericolic fat stranding [11,14].

As clinical presentation and complementary diagnostic studies are routinely non-specific and suggestive of inflammatory bowel disease, final proper diagnosis is frequently delayed until surgical resection of the affected areas is performed and pathological examination of the surgical specimens is performed, making adequate preoperative diagnosis rare [15]. Macroscopic findings consist of thickened intestinal walls with cobblestone mucosa after fixation (Figure 5), while microscopic examination usually discloses arterial-sparing intimal smooth muscle hyperplasia of small and medium-sized mesenteric veins, non-thrombotic obliteration of the vessels, mucosal ischemia, and adipose tissue necrosis of the mesocolon (Figure 6).

As an adequate final diagnosis of IMHMV is a challenge and surgical treatment is usually delayed until symptoms become refractory or complications occur, unnecessary medical treatment with immunomodulators, antibiotics, steroids, biologic agents, 5-aminosalicylates, and other medical therapies for inflammatory bowel conditions are often employed [7,12,15,16]. Alongside unnecessary medical treatments and their potential side effects, delayed adequate treatment with surgical resection may lead to severe and life-threatening complications, such as rectal bleeding. Our patient developed early massive bleeding less than a month after initial symptoms, an infrequent clinical presentation of IMHMV.

**Figure 8:**
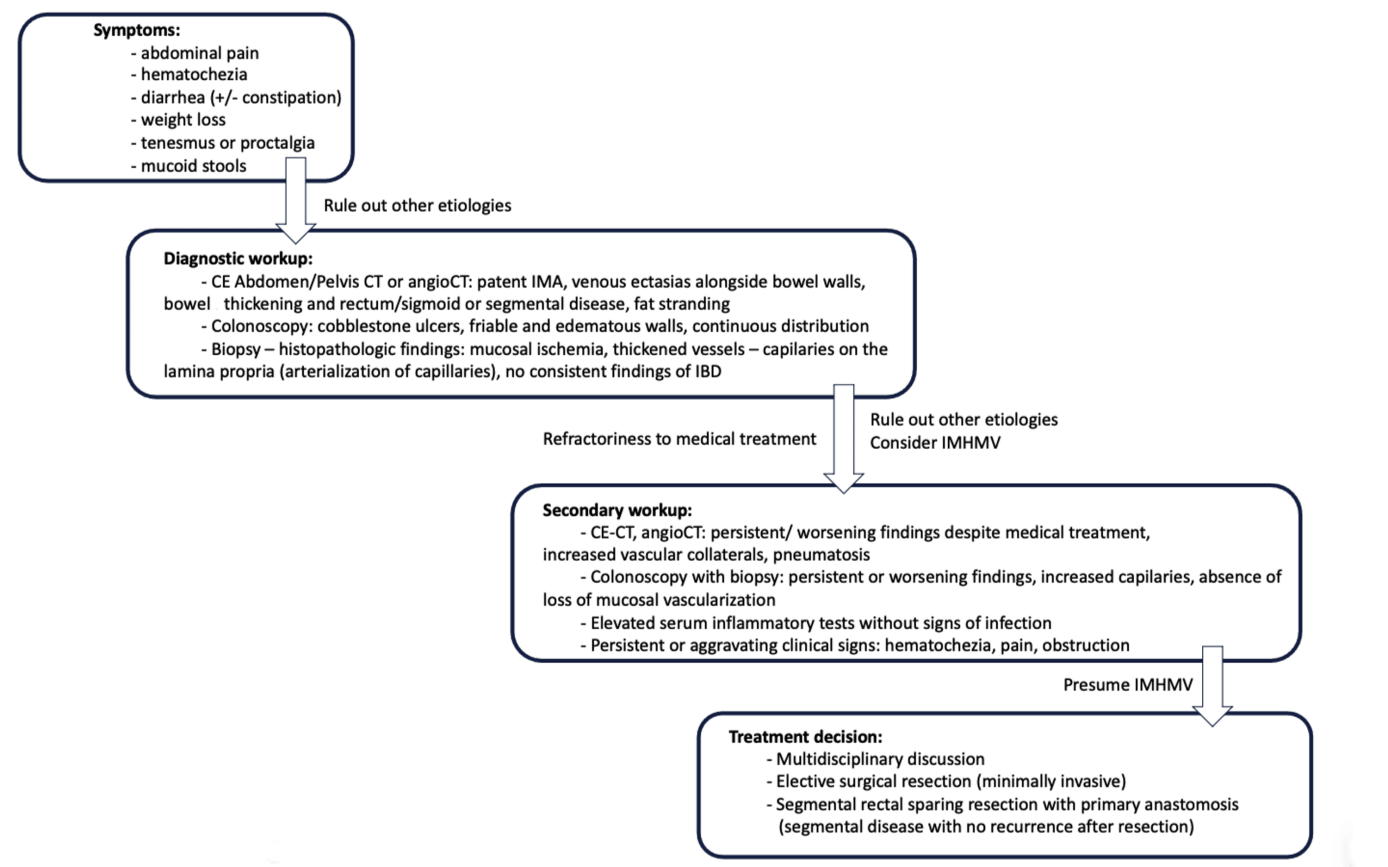
Algorithm for workup and treatment of a patient with suspected IMHMV. IMHMV: Idiopathic myointimal hyperplasia of the mesenteric veins; CE: contrast-enhanced; CT: computed tomography; IMA: inferior mesenteric artery; IBD: inflammatory bowel disease

Although there is no definitive preoperative test for IMHMV as it is an extramural disease, and mucosal biopsies obtained by colonoscopy are not conclusive, there are few reports of IMHMV diagnosed (or highly suspected) before surgical treatment avoiding unnecessary treatment or potentially fatal complications such as massive bleeding, which occurred in our patient [4,15]. Alongside the awareness of the disease and a high index of suspicion that is crucial for early diagnosis, careful interpretation of clinical information, image studies, and biopsies may allow proper preoperative diagnosis. We propose a workup algorithm for patients with possible IMHMV in Figure 8.

## Conclusions

Idiopathic myointimal hyperplasia of the mesenteric veins is a rare cause of intestinal ischemia. Careful interpretation of image studies, generous acquisition of biopsies by colonoscopy, and awareness of the differential diagnoses of IMHMV in cases suggestive of segmental ischemia may avoid unnecessary medical treatment and life-threatening complications before proper surgical treatment.
